# Geographic differences in allele frequencies of susceptibility SNPs for cardiovascular disease

**DOI:** 10.1186/1471-2350-12-55

**Published:** 2011-04-20

**Authors:** Keyue Ding, Iftikhar J Kullo

**Affiliations:** 1 Division of Cardiovascular Diseases, Mayo Clinic, Rochester MN 55905, USA

**Keywords:** cardiovascular disease, genetics, genome-wide association study, risk allele frequency, population differentiation

## Abstract

**Background:**

We hypothesized that the frequencies of risk alleles of SNPs mediating susceptibility to cardiovascular diseases differ among populations of varying geographic origin and that population-specific selection has operated on some of these variants.

**Methods:**

From the database of genome-wide association studies (GWAS), we selected 36 cardiovascular phenotypes including coronary heart disease, hypertension, and stroke, as well as related quantitative traits (eg, body mass index and plasma lipid levels). We identified 292 SNPs in 270 genes associated with a disease or trait at *P *< 5 × 10^-8^. As part of the Human Genome-Diversity Project (HGDP), 158 (54.1%) of these SNPs have been genotyped in 938 individuals belonging to 52 populations from seven geographic areas. A measure of population differentiation, *F*_ST_, was calculated to quantify differences in risk allele frequencies (RAFs) among populations and geographic areas.

**Results:**

Large differences in RAFs were noted in populations of Africa, East Asia, America and Oceania, when compared with other geographic regions. The mean global *F*_ST _(0.1042) for 158 SNPs among the populations was not significantly higher than the mean global *F*_ST _of 158 autosomal SNPs randomly sampled from the HGDP database. Significantly higher global *F*_ST _(*P *< 0.05) was noted in eight SNPs, based on an empirical distribution of global *F*_ST _of 2036 putatively neutral SNPs. For four of these SNPs, additional evidence of selection was noted based on the integrated Haplotype Score.

**Conclusion:**

Large differences in RAFs for a set of common SNPs that influence risk of cardiovascular disease were noted between the major world populations. Pairwise comparisons revealed RAF differences for at least eight SNPs that might be due to population-specific selection or demographic factors. These findings are relevant to a better understanding of geographic variation in the prevalence of cardiovascular disease.

## Background

Although ethnic and geographic differences in the rates of cardiovascular disease are well known [1,2], the basis for such differences is not fully understood and the possible contribution of genetic factors has not been investigated. Genome-wide association studies (GWAS) have led to the identification of multiple genetic susceptibility variants (mostly single nucleotide polymorphisms, SNPs) for common diseases, such as atherosclerotic vascular disease [3]. However, most of the susceptibility SNPs were identified from populations of European ancestry. It is not known whether such SNPs also mediate susceptibility in other populations, whether the risk allele frequencies (RAFs) of these SNPs differ among the global populations, and whether these differences are due to forces of population-specific selection (ie, local selection).

An evolutionary perspective might explain why contemporary humans are at high risk for atherosclerotic vascular disease and help to better understand geographic variation in disease susceptibility [4]. Since geographically separated populations might be subject to distinctive selective environments, population-specific selection can increase population differentiation at the selected locus. However, large differences in RAFs between geographic regions are more likely to have resulted from genetic drift during population expansion after a bottleneck rather than by natural selection, a phenomenon also known as 'allelic surfing' [5-7]. From a genetic epidemiology perspective, it is of interest to explore whether RAF differences exist for susceptibility variants for cardiovascular disease, and if so, whether the differences can be accounted by allelic surfing or by natural selection.

In order to elucidate the evolutionary mechanisms that may have influenced the history and spread of genes/variants associated with cardiovascular disease and related risk factors, we calculated differences in RAFs within and out of a region of interest, and tested the population differentiation of SNPs identified in GWAS as influencing risk of cardiovascular disease. We hypothesized that SNPs mediating susceptibility to cardiovascular disease differ in frequency between human populations and may show evidence for population-specific selection.

## Methods

### GWAS of cardiovascular disease phenotypes and related quantitative traits

The GWAS database [8] (accessed on Sep. 6^th ^2009) includes 211 distinct disease and traits, as well as >1,500 associated SNPs. From this database, we selected 36 cardiovascular diseases and traits, including coronary heart disease, stroke, and hypertension, and quantitative traits (ie, body mass index blood pressure and plasma lipid levels). A detailed description of selection of the cardiovascular disease phenotypes and related quantitative traits is provided in the Additional file 1. We identified 292 SNPs in 270 genes associated with a disease or trait at *P *< 5 × 10^-8^. The risk allele was ascertained from original reports, eg, the allele associated with either an odds ratio >1 for disease phenotypes or with higher levels of body mass index, low-density lipoprotein (LDL) cholesterol, triglycerides and blood pressure, and lower levels of high-density lipoprotein (HDL) cholesterol.

### Genotype data

For our population genetics analyses, we obtained genotype data from the Human Genome Diversity Project (HGDP) [9], as part of which 642,690 SNPs were genotyped in 938 unrelated individuals from 52 populations [10]. The 52 populations belong to seven geographic areas: African, Middle East, Europe, Central and South Asia, East Asia, America, and Oceania (Additional file 2). Of 292 SNPs, genotype data for 158 SNPs (54.1%) were available in the HGDP database and analyzed in the present study (Additional file 3).

### Statistical analysis

We calculated the global and regional risk allele frequency (RAF) for each SNP. For risk allele *i *of each SNP, we computed the average RAF () within each geographic area *j*, as well as the difference with the average RAF computed over all other populations as , where  is the average RAF of allele *i *in all populations not belonging to the geographic region *j*. We used the method of Hofer et al. [6] to randomly permute populations (10,000 times) between geographic areas and recomputed *ΔF *each time, to obtain its null distribution and test for the significance of Δ*F *for each risk allele.

The measure of population differentiation (*F*_ST_) was calculated as described by Weir [11], using a Perl script that we had previously developed [12]. We calculated the global *F*_ST _among 52 populations and this served as a summary of the global population differentiation. In addition, the *F*_ST _among the seven geographical areas (7 × 7 matrices, populations in the same geographical area were combined), and pairwise *F*_ST _in any two populations (52 × 52 matrices) were calculated. In order to test whether the mean *F*_ST _of the 158 susceptibility SNPs was significantly different from that of random markers, we selected 158 random SNPs with allele frequency distribution similar to that of 158 susceptibility SNPs 1,000 times. The mean *F*_ST _of the 158 susceptibility SNPs was compared to the distribution of mean *F*_ST _of the 158 random SNPs.

To assess the statistical significance of differences in *F*_ST _across populations, an empirical distribution of *F*_ST _was estimated by selecting 2036 autosomal markers from HGDP database. The 2036 random markers located within intergenic regions, were selected based on the annotation tables of human chromosomes (http://genome.ucsc.edu). These regions were separated by at least 1 MB from the closest exon, did not include centromeric regions, and provide an appropriate null distribution for spatial patterns of variation expected for putatively neutral regions [12]. Each *F*_ST _value was compared to the corresponding empirical distribution of *F*_ST _values to generate a *P *value, which was corrected for minor allele frequency by comparing only to SNPs (*n *= 2036) from the empirical distribution that fell into the same minor allele frequency bin (*P*cor) [13]. For SNPs with a significantly higher global *F*_ST_, we also obtained the integrated Haplotype Score (iHS) statistic using the HGDP selection browser [14,15]. The iHS statistic is based on the differential levels of LD surrounding a positively selected allele compared to a background allele at the same position [16].

We assessed the geographic patterns of pairwise *F*_ST _based on geographic distance and human migration routes. The method for calculating geographic distance for each pair of populations is described further in Additional file 1. A linear regression coefficient was calculated from the scatterplot of pairwise *F*_ST _against geographic distances.

## Results

### Distribution of risk allele frequencies (RAFs)

**Figure 1:**
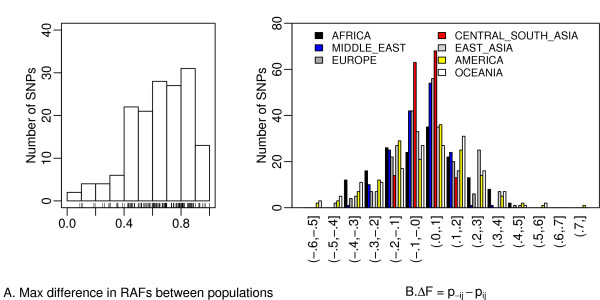
**Distribution of risk allele frequency (RAF) in HGDP populations**. A. Differences in RAFs between any 2 of the 52 populations in the HGDP panel for the 158 SNPs associated with cardiovascular-diseases traits. B. Differences of RAF () for the comparison of a given tested region (eg, Africa) versus the rest of the world. Negative Δ*F *indicates that the risk allele has a higher frequency within the tested population than in the rest of the world (see methods section for the denotation of  and ).

We first characterized the distribution of RAFs for susceptibility SNPs for cardiovascular disease and related risk factors. The RAFs varied significantly among the populations, from being either 'fixed' (RAF = 1) or 'missed' (RAF = 0). A summary of the maximum differences of RAFs (ie, the largest difference of all the pairwise population comparisons) between any 2 of the 52 populations for each SNP is presented in Figure 1A. Seventy-two risk alleles were 'fixed' in at least one population whereas 86 risk alleles were 'missed' in at least one population.

We then determined the prevalence of large RAF differences between geographic regions. We tested whether populations belonging to the same geographic area have more similar RAFs than expected by chance, either due to shared demographic history or due to shared selective events, as suggested by Hofer et al. [6]. Figure 1B summarizes the distribution of differences of the average of RAFs in one of the tested geographic areas, as well as the differences with the average RAFs computed over all other populations (ie, ). A negative Δ*F *indicates that the RAF is higher within the tested geographic area than in the rest of the world. We used an arbitrary threshold (Δ*F *> 0.3 or Δ*F *< -0.3) to define a large RAF difference [6]. Large differences in RAF between geographic areas were frequent: 69 out of 158 (43.7%) risk alleles have Δ*F *> 0.3 or Δ*F *< -0.3 for at least one comparison. Of the seven geographic regions, Africa, East Asia, America, and Oceania showed the largest number of loci with large RAF differences (Additional file 4). As expected under the out-of-Africa hypothesis, the differences in RAF are strikingly large when comparing Africa with the remaining geographic regions. The largest observed Δ*F *(0.731) was found between American and non-American populations for the SNP 'rs174570' in *FADS2 *(a gene that regulates unsaturation of fatty acids); the RAF in the native American population (0.048) was much lower than in the remaining populations (0.779).

### Global population differentiation

**Figure 2:**
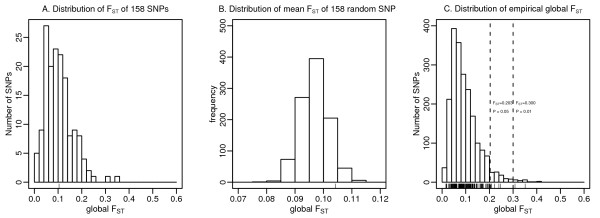
**Distribution of *F***_**ST**_. A. Distribution of *F*_ST _of 158 SNPs associated with cardiovascular diseases and related intermediate traits. The vertical bar shows the mean of global *F*_ST _of these 158 SNPs (*F*_ST _= 0.1042). B. Distribution of empirical global mean *F*_ST _of 158 autosomal SNPs randomly sampled from HGDP database 1000 times. The distribution of minor allele frequency of the randomly selected 158 SNPs was matched to the observed 158 SNPs. The vertical bar shows the mean of global *F*_ST _of 158 SNPs associated with cardiovascular diseases and related intermediate traits (*F*_ST _= 0.1042). C. Distribution of empirical global *F*_ST _of 2,036 markers assumed to be neutral, genotyped in 938 unrelated individuals form HGDP. Two vertical lines indicate 95% and 99% percentile of the global *F*_ST _distribution. The vertical bars show the values of global *F*_ST _value of 158 susceptibility SNPs in the present study.

**Figure 3:**
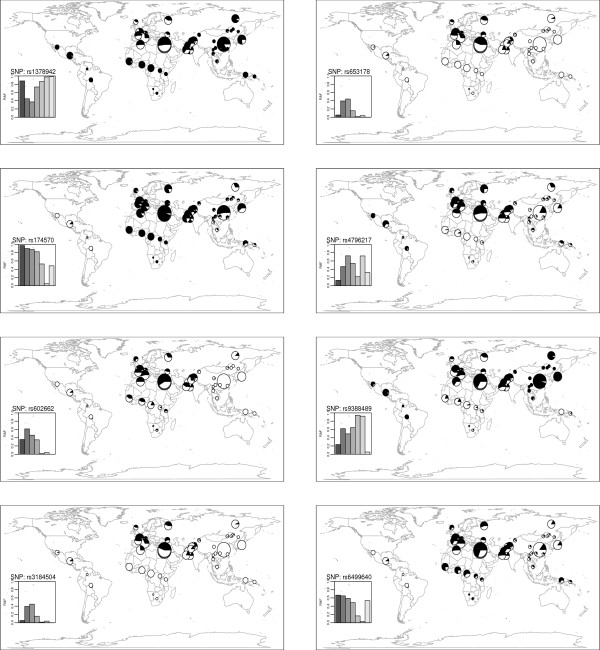
**Geographical distribution of risk (black) and wild-type (white) alleles in eight SNPs with a significantly higher *F***_**ST **_**among the 52 populations**. In each subplot, the radius of a pie chart represents the sample size in a given population. The inserted barplot shows the average frequency of the risk allele among the seven geographic regions. From left to right, the bars indicate Africa, Middle East, Europe, Central South Asia, East Asia, America, and Oceania, respectively.

We calculated the global *F*_ST _as a measure of allele frequency differences across the 52 populations (Figure 2A). The mean global *F*_ST _for the 158 cardiovascular susceptibility SNPs was 0.1042, not significantly larger than the mean global *F*_ST _of 158 randomly sampled SNPs (*P *= 0.064) (Figure 2B), indicating that overall, the 158 SNPs associated with cardiovascular diseases were not more differentiated than random markers. We assessed the statistical significance for each susceptibility SNP by comparing its global *F*_ST _with the empirical distribution of global *F*_ST _for 2036 autosomal intergenic SNPs from the HGDP database (Figure 2C) [12]. Among 158 SNPs, eight SNPs had a statistically significantly higher *F*_ST _(*F*_ST _> 0.203; *P *< 0.05) (Table 1) even after correcting for allele frequency. Three of these SNPs were associated with diastolic blood pressure (rs1378942 in *CSK*, rs653178 in *ATXN2 *and rs3184504 in *SH2B3*), two with type 1 diabetes (rs9388489 in *C6orf97 *and rs3184504 in *SH2B3*), as well as one each with a lipid trait (rs174579 in *FADS2*), body mass index (rs6499640 in *FTO*), folate pathway (rs602662 in *FUT2*) and inflammation (rs4796217 in *CCL4L2*). The world maps in Figure 3 show significant variation in the geographical distributions of the RAFs of eight SNPs with a significantly higher global *F*_ST_. Correction for minor allele frequency led to another four SNPs (rs17696736, rs2237892, rs7578597, and rs673548) reaching statistical significance (Additional file 5).

**Table 1:** A list of SNPs with significantly higher global *F*_ST _(*P *< 0.05)

SNPs	Trait	Gene	RAF	Global *F*_ST_	*P *value	*P*_*cor*_
rs1378942	Diastolic blood pressure [17]	*CSK*	0.708 (G)	0.247	0.025	0.022
rs653178	Diastolic blood pressure [17]	*ATXN2*	0.177 (G)	0.205	0.049	0.031
rs174570	Total, HDL and LDL cholesterol [36]	*FADS2*	0.730 (C)	0.351	0.002	0.000
rs4796217	Macrophage-mediated inflammation [37]	*CCL4L2*	0.425 (T)	0.221	0.040	0.032
rs602662*	Folate pathway [22,38]	*FUT2*	0.294 (A)	0.240	0.027	0.028
rs9388489	Type 1 diabetes [39]	*C6orf97*	0.634 (G)	0.308	0.008	0.002
rs3184504*	Type 1 diabetes [39]; diastolic blood pressure and systolic blood pressure[17]	*SH2B3*	0.179 (T)	0.210	0.045	0.031
rs6499640	BMI and weight[40]	*FTO*	0.455 (A)	0.208	0.045	0.037

RAF: risk allele frequency; *P*_cor_, *P *value after correcting for minor allele frequency; *CSK*, c-src tyrosine kinase; *ATXN2*, ataxin 2; *FADS2*, fatty acid desaturase 2; *CCL4L2*, chemokine (C-C motif) ligand 4-like 2; *FUT2*, fucosyltransferase 2; *C6orf97*, chromosome 6 open reading frame 173; *SH2B3*, SH2B adaptor protein 3; *FTO*, fat mass and obesity associated

*, non-synonymous SNPs

We also plotted the patterns of pairwise *F*_ST _for these eight SNPs in any two of 52 populations and any two of seven geographic areas (Additional file 6). The patterns of population differentiation revealed the specific pattern of frequency difference of each disease-susceptibility variant among the world's populations. For example, the low RAF of rs174570 in *FADS2 *(associated with LDL, HDL and total cholesterol and triglycerides) in the indigenous populations of America (0.038) might contribute to the significant population differentiation between America and any of the populations in the other six geographic regions (ie, 393 (29.6%) pairwise comparisons showed a significantly higher *F*_ST_). The three SNPs (ie, rs1378942, rs653178, and rs3184504) associated with blood pressure [17] showed different patterns of population differentiation: RAFs in rs1378942 were > 0.5 in most geographic regions (except in Middle East and Europe); whereas RAFs in rs653178 and rs3184504 were relatively higher in Middle East and Europe compared to the remaining geographic regions (<0.1). Pairwise comparisons (in any two out of seven geographic areas) revealed differences in RAFs for more than half of the SNPs (84 out of 158) that might be due to genetic drift or local selection. The number of genes that showed a significantly higher *F*_ST _in pairwise comparisons among seven geographic areas is shown in Additional file 7. The results indicate that for risk alleles, East Asia is more differentiated from other geographic areas. Furthermore, in comparing any two of the 52 populations, for each SNP, at least one pairwise population showed a significantly higher pairwise *F*_ST_. The most undifferentiated SNP among the 52 populations was rs6511720 in *LDLR*, the RAF (0.921) being relatively high worldwide, as discussed above.

### Geographic patterns of population differentiation

Genetic differentiation increases with geographic distance [18], and we therefore tested whether the *F*_ST _(as a measure of genetic 'distance') is correlated with the geographic distance. For each SNP, a linear regression analysis between genetic distances and geographic distances between populations was performed. When combining data from 158 SNPs, the coefficient of correlation (*R*^2^) was 0.271 (*P *< 2.2 × 10^-16^). When each SNP was analyzed separately, all but five SNPs [ie, rs13194491, rs2383208, rs2476601, rs4900384, and rs693] showed a correlation between genetic 'distance' and geographic distance.

### iHS statistic

The iHS statistic is based on the differential levels of LD surrounding a positively selected allele compared to a background allele at the same position [16]. Voight et al. [16] defined iHS < -1.5 or iHS > 1.5 as suggestive evidence for natural selection, and iHS < -2 or iHS > 2 as strong evidence for selection for variants that have not yet reached fixation. Using the HGDP selection browser [14,15], we found that of the eight SNPs listed in Table 1, rs1378942 showed strong evidence for selection (iHS = 2.11 in America and iHS = 2.09 in Oceania); and three SNPs showed suggestive evidence of natural selection (rs653178, iHS = 1.53 in Europe; rs9388489, iHS = 1.97 in Oceania; and rs3184504, 1.99 in Europe and 1.83 in Middle East).

## Discussion

In the present study we used genotype data from 52 populations in the Human Genome Diversity Project (HGDP) to characterize the worldwide patterns of risk allele frequencies (RAFs) of 158 common SNPs associated with cardiovascular diseases and related quantitative traits. Our null hypothesis was that there is no variation of RAFs of SNPs associated with cardiovascular diseases and intermediate phenotypes, among the populations. Out of 158 susceptibility SNPs, substantial variations in RAFs of such SNPs were noted among the 52 populations, including some risk alleles being fixed or missed in at least one population. In addition we found that eight SNPs showed significant differences of RAFs among the seven geographic areas. These findings provide insights into the 'global' genetic epidemiology of cardiovascular disease.

Maximum differences in RAFs between any 2 populations ranged from 0.089 to 1.000 across SNPs with a mean of 0.661 (Figure 1A). In comparison with the rest of world, large differences in RAFs (ie, Δ*F *> 0.3 or Δ*F *< -0.3) were noted for the regions of Africa, East Asia, America, and Oceania (Additional file 4 and Figure 1B), consistent with the out-of-Africa hypothesis. Several explanations can be put forth for the larger differences in RAFs, including either demographic changes or shared selective events. Hofer et al. [6] suggested that large allele frequency differences between human continental groups are more likely to have occurred by genetic drift during population expansion after a bottleneck, than by selection. Using all the 158 SNPs sampled from the HGDP database, we also calculated Nei's genetic distances by 'dist.genet' function in the 'ade4' package [19] in *R *and then used multidimensional scaling (MDS) (by 'cmdscale' function in R) to assess population differences (Additional file 8). The MDS shows differentiation among the populations corresponding to the three main clusters (ie, Europeans, Africans, and Asians). Analysis of molecular variance (AMOVA) [20] using the 'amova' function in the 'pegas' package [21] in *R *showed that, of the total variance, 28.6% was due to variance among the seven geographic regions, 18.4% was due to variance among the populations within geographic regions, and the remaining (53.0%) was due to variance among individuals within populations (Additional file 9).

The susceptibility variants analyzed in the present study are likely functional variants (or in linkage disequilibrium with the causal variants). The large differences in RAFs might be due to either natural selection or population demography. For example, the non-synonymous SNP rs602662 within *FUT2 *is in strong linkage disequilibrium with a non-sense mutation (rs601338), a plausible causal variant [22]. SNP rs3184504 is located in exon 3 of *SH2B3 *which encodes the T-cell adapter protein LNK [23] and might be a causal variant (Table 1). The association of the other six SNPs has been replicated in multiple cohorts or independent samples, suggesting they are likely to be 'true' associated loci. We found additional evidence for recent positive selection (based on iHS) [16] in four (rs1378942, rs653178, rs9388489, and rs3184504) of these eight SNPs. An elevated iHS score suggests that the ancestral allele itself or the selected allele hitchhiking with the ancestral allele may be the target of selection. The iHS for each of the four SNPs was positive, indicating that the ancestral allele was under selection.

We did not observe a significantly higher degree of population differentiation for cardiovascular disease susceptibility SNPs identified in GWAS (Figure 2B). The mean global *F*_ST _of the 158 SNPs (0.1042) was not significantly higher than the *F*_ST _for random markers. Lohmueller et al. [24] found that 48 SNPs associated with common diseases were not significantly more differentiated across populations than random SNPs, and in another study of 25 disease-associated SNPs identified in GWAS, the mean global *F*_ST _(0.100) was not significantly higher than random SNPs in the genome [13]. Using *F*_ST _and iHS, Southam et al. [25] did not find consistent patterns of selection to confirm the 'thrifty-genotype' hypothesis for metabolic syndrome/diabetes based on HapMap data.

It should be noted that, after correction for multiple comparison either by Bonferroni method or false discovery rate, only one SNP (rs174540) remained significant. Therefore population history and demography are likely to explain most of the difference in RAFs among populations. However, in previous studies, signatures of natural selection have also been noted in *FTO *(iHS = 1.991) [25], *FUT2 *[26], *ATXN2 *(high levels of LD) [27], and *SH2B3 *(iHS = -2.02 for SNP rs3184504) [15]. The non-synonymous SNP rs3184504 in *SH2B3 *associated with higher diastolic blood pressure (minor allele 'T') may be under recent positive selection [17]. In the HapMap samples, this derived T allele has been shown to occur on a long haplotype (~1.5 MB) (iHS = -2.76, *P *< 0.006) [16], and local selection was noted (ie, *F*_ST _= 0.260 for CEU-YRI comparison and *F*_ST _= 0.290 for CEU-JPT/CHB comparison). The present analysis confirmed that in the HGDP sample, significant population differentiation (*F*_ST _= 0.207, *P *= 0.048) could be attributed to a relatively higher RAF in the Middle East and Europe. A high global *F*_ST _for rs7901695 of *TCF7L2 *(0.361) was noted in the HapMap samples [25], but not in the HGDP sample (0.188), possibly related to differences in sample selection between HapMap and HGDP.

Coop et al. [28] examined the role of geography and population history in the spread of selectively favored alleles, using the HapMap and HGDP databases, and argued that strong, sustained selection that drives alleles from low frequency to near fixation has been relatively rare during the past ~70,000 years [28]. The importance of geography on patterns of genetic variation has been established in previous studies [9,29-31]. We examined the prevalence of large RAFs between geographic regions and noted that RAFs of three SNPs with a significantly higher global *F*_ST _are quite low (high)/even missed (fixed) in several populations (Figure 3). Spatial and/or temporal variation of selective pressures, such as pathogens, climate or diet, may have restricted local selection to particular populations or environments [32]. The 'ancestral-susceptibility' hypothesis for common 'complex' diseases [33] states that the ancestral allele is maladaptive in the modern environment and associated with increased disease susceptibility. We found that the ancestral allele was the risk allele in 65 out of 158 SNPs (41.1%) based on dbSNPs and UCSC database. Thus, a subset of the cardiovascular disease susceptibility SNPs conforms to the hypothesis of 'ancestral susceptibility model' for common 'complex' diseases.

Geographic variation in the prevalence of phenotypes of medical relevance can partly be due to differential RAFs among different populations. Levels of total and LDL cholesterol in the Pima Indians (living in central and southern Arizona and Sonora (Mexico)) are lower than in populations of European origin [34]. The risk allele of rs174570 in *FADS2 *(a gene that regulates unsaturation of fatty acids) is missed in Pima (RAF = 0.000), but fixed in Africans (RAF = 1) (Figure 3), raising the possibility that the low RAFs of SNPs may contribute to the low LDL cholesterol levels in Pima Indians. Pairwise comparison of RAFs among 52 populations and seven geographic areas indicated a high population differentiation between American and non-Americans, consistent with a previous study [35] showing Native Americans have greater differentiation than populations from other continental regions.

Several limitations of our study need to be mentioned. There is a potential for bias in selecting SNPs from the GWAS database as well as from the HGDP database. The SNPs were selected based on GWAS in populations of European ancestry and we were not able to characterize the patterns of geographic difference of RFs for SNPs ascertained in other specific populations, such as Africans and Asians. In addition, the majority of the HGDP populations are poorly represented in the genotyping chips and only 158 out of 292 SNPs have been genotyped in HGDP [9]. The genotyping platforms used in published GWAS varied and there was no standard threshold in declaring significant hits. Ascertainment bias in Oceania should be noted since only two populations (Papuan and NAN Melanesian) were sampled in this geographic region. Nonetheless the present study highlights a novel approach to understanding the global genetic epidemiology of cardiovascular disease, the leading cause of death worldwide and is also a step towards understanding the evolutionary genetics of this disease.

## Conclusions

In conclusion, large differences in common SNPs that influence risk of cardiovascular disease were noted among the worldwide populations and are mostly due to genetic drift. The global mean *F*_ST _for these SNPs did not differ significantly from random variants in the genome. However, pairwise comparisons revealed differences in RAFs in eight SNPs that might be due to local natural selection or demographic factors. These findings may help to better understand geographic variation in the prevalence of cardiovascular disease.

## Abbreviations

SNP: single nucleotide polymorphism; RAF: risk allele frequency; GWAS: genome-wide association study;

## Competing interests

The authors declare that they have no competing interests.

## Authors' contributions

Conception and Design: KD and IJK; Data analyses: KD; Manuscript preparation: KD and IJK. Both authors have given final approval of the manuscript.

## Pre-publication history

The pre-publication history for this paper can be accessed here:

http://www.biomedcentral.com/1471-2350/12/55/prepub

## Supplementary Material

Additional file 1 **Supplementary methods**. The supplementary methods describe 1) selection of cardiovascular disease phenotypes and related quantitative traits; and 2) calculation of geographic distance in kilometers for each pair of populations. Click here for file

Additional file 2 **Table S1**. Sample size in the HGDP populations. Click here for file

Additional file 3 **Table S2**. A list of SNPs associated with cardiovascular diseases/traits identified in genome-wide association studies. Click here for file

Additional file 4 **Table S3**. The number of risk alleles with various categories of RAF differences: comparisons of a given geographic area versus the rest of the world. Click here for file

Additional file 5 **Table S4**. A list of SNPs with significantly higher global *F*_ST _(*Pcor *< 0.05) Click here for file

Additional file 6 **Figure S1**. Pairwise comparison of population differentiation for SNPs with a significantly higher global *F*_ST _among 52 populations. The shaded boxes in the matrices indicate the significance level of *F*_ST _based on the empirical distribution of the 2,036 SNPs for each pair of populations. The inserted subplot shows the comparison for the seven populations based on the geographic areas. Click here for file

Additional file 7 **Figure S2**. Number of SNPs that showed a significantly higher *F*_ST _in pairwise comparisons among the seven geographic areas. Click here for file

Additional file 8 **Figure S3**. Multidimensional scaling analysis plot (dimension I/II) of 938 individuals from seven geographic areas using 158 SNPs sampled from the HGDP database. Description: The population 'Mozabite' (black circle) was clustered into the 'Middle East', and 'Europe', which was geographically located in North Africa. The populations in 'Central_South_Asia' showed a cline from 'Middle East' and 'Europe' to 'East_Asia'. Different colors and point characters indicate seven geographic areas (see figure legend). Click here for file

Additional file 9 **Table S5**. Analysis of molecular variance (AMOVA) of 158 SNPs. Click here for file
