# Supplementary material for: Geographic differences in allele frequencies of susceptibility SNPs for cardiovascular disease
Source: BMC Med Genet. 2011 Apr 20;12:55. doi: 10.1186/1471-2350-12-55 (PMC3103418; doi:10.1186/1471-2350-12-55)
Supplement: Additional file 1 — Supplementary methods. The supplementary methods describe 1) selection of cardiovascular disease phenotypes and related quantitative traits; and 2) calculation of geographic distance in kilometers for each pair of populations. [file 1471-2350-12-55-S1.DOC]

**Additional File 1: Supplementary Methods**

***Selection of cardiovascular disease phenotypes and related quantitative traits***

We have categorized phenotypes into cardiovascular diseases and related risk factors based on our recently published review article [1]. The diseases phenotypes include coronary artery disease, peripheral arterial disease, and abdominal aortic aneurysm, whereas the related risk factors include obesity, Type 2 diabetes, hypertension, plasma lipid levels, and circulation markers of inflammation. We reviewed the 211 unique disease and traits in the catalogue of GWA studies ([www.genome.gov/GWAstudies](http://www.genome.gov/GWAstudies)) and included 36 phenotypes in Table S2.

***Calculation of geographic distance in kilometers for each pair of populations***

We calculated geographic distance in kilometers for each pair of populations based on great circle distances using the haversine [2], and taking into account the routes of human migration. The coordinates (latitude, longitude) for each population were obtained from Cann [3]. For populations where ranges of coordinates were provided, the mean of the latitudes and the mean of the longitudes of the reported regions were used to characterize the population’s location. Five obligatory waypoints [Anadyr, Russia (64N, 177E); Cairo, Egypt (30N, 31E); Istanbul, Turkey (41N, 28E); Phnom Penh, Cambodia (11N, 104E); and Prince Rupert, Canada (54N, 130W)] were used so that the distance between two points is the sum of the great circle distances between the points and the waypoint(s) in the path connecting them, and the great circle distance(s) between waypoints if two or more waypoints are needed [4]. Waypoints were used to make our between-continent distance estimates more reflective of human migration patterns [4].

**References**

1. Ding K, Kullo IJ: **Genome-wide association studies for atherosclerotic vascular disease and its risk factors**. *Circ Cardiovasc Genet* 2009, **2**(1):63-72.

2. Sinnott RW: **Virtues of the Haversine**. *Sky Telescope* 1984, **68**:159-161.

3. Cann HM, de Toma C, Cazes L, Legrand MF, Morel V, Piouffre L, Bodmer J, Bodmer WF, Bonne-Tamir B, Cambon-Thomsen A *et al*: **A human genome diversity cell line panel**. *Science* 2002, **296**(5566):261-262.

4. Ramachandran S, Deshpande O, Roseman CC, Rosenberg NA, Feldman MW, Cavalli-Sforza LL: **Support from the relationship of genetic and geographic distance in human populations for a serial founder effect originating in Africa**. *Proc Natl Acad Sci U S A* 2005, **102**(44):15942-15947.
