# Supplementary material for: Geographic differences in allele frequencies of susceptibility SNPs for cardiovascular disease
Source: BMC Med Genet. 2011 Apr 20;12:55. doi: 10.1186/1471-2350-12-55 (PMC3103418; doi:10.1186/1471-2350-12-55)
Supplement: Additional file 2 — Table S1. Sample size in the HGDP populations. [file 1471-2350-12-55-S2.DOC]

| **Table S1**. Sample size in the HGDP populations. | | | | | |
| --- | --- | --- | --- | --- | --- |
| **Population** | **Geo_Area** | **Sample** | **Population** | **Geo_Area** | **Sample** |
| Bantu_NE | AFRICA | 11 | Hezhen | EAST_ASIA | 9 |
| Bantu_SouAfr | AFRICA | 8 | Japanese | EAST_ASIA | 28 |
| Biaka_Pygmy | AFRICA | 22 | Lahu | EAST_ASIA | 8 |
| Mandenka | AFRICA | 22 | Miaozu | EAST_ASIA | 10 |
| Mbuti_Pygmy | AFRICA | 13 | Mongola | EAST_ASIA | 10 |
| Mozabite | AFRICA | 27 | Naxi | EAST_ASIA | 8 |
| San | AFRICA | 5 | Oroqen | EAST_ASIA | 9 |
| Yoruba | AFRICA | 21 | She | EAST_ASIA | 10 |
| Colombian | AMERICA | 7 | Tu | EAST_ASIA | 10 |
| Karitiana | AMERICA | 13 | Tujia | EAST_ASIA | 10 |
| Maya | AMERICA | 21 | Xibo | EAST_ASIA | 9 |
| Pima | AMERICA | 14 | Yakut | EAST_ASIA | 25 |
| Surui | AMERICA | 8 | Yizu | EAST_ASIA | 10 |
| Balochi | CENTRAL_SOUTH_ASIA | 24 | Adygei | EUROPE | 17 |
| Brahui | CENTRAL_SOUTH_ASIA | 25 | Basque | EUROPE | 24 |
| Burusho | CENTRAL_SOUTH_ASIA | 25 | French | EUROPE | 28 |
| Hazara | CENTRAL_SOUTH_ASIA | 22 | from_Bergamo | EUROPE | 12 |
| Kalash | CENTRAL_SOUTH_ASIA | 23 | Orcadian | EUROPE | 15 |
| Makrani | CENTRAL_SOUTH_ASIA | 25 | Russian | EUROPE | 25 |
| Pathan | CENTRAL_SOUTH_ASIA | 22 | Sardinian | EUROPE | 28 |
| Sindhi | CENTRAL_SOUTH_ASIA | 24 | Tuscan | EUROPE | 7 |
| Uygur | CENTRAL_SOUTH_ASIA | 10 | Bedouin | MIDDLE_EAST | 45 |
| Cambodian | EAST_ASIA | 10 | Druze | MIDDLE_EAST | 42 |
| Dai | EAST_ASIA | 10 | Palestinian | MIDDLE_EAST | 46 |
| Daur | EAST_ASIA | 9 | NAN_Melanesian | OCEANIA | 11 |
| Han | EAST_ASIA | 44 | Papuan | OCEANIA | 17 |
