# Supplementary material for: Geographic differences in allele frequencies of susceptibility SNPs for cardiovascular disease
Source: BMC Med Genet. 2011 Apr 20;12:55. doi: 10.1186/1471-2350-12-55 (PMC3103418; doi:10.1186/1471-2350-12-55)
Supplement: Additional file 3 — Table S2. A list of SNPs associated with cardiovascular diseases/traits identified in genome-wide association studies. [file 1471-2350-12-55-S3.DOC]

| **Table S2**. A list of SNPs associated with cardiovascular diseases/traits identified in genome-wide association studies | | | |
| --- | --- | --- | --- |
| **No.** | **SNPs** | **Disease or Trait** | **Reference** |
| 1 | rs1004446 | Type 1 diabetes | [1] |
| 2 | rs10096633 | Triglycerides | [2] |
| 3 | rs10468017 | Serum HDL cholesterol | [3] |
| 4 | rs10494366 | QT interval prolongation | [4] |
| 5 | rs10495809* | Hypertension (early onset) | [5] |
| 6 | rs10509540 | Type 1 diabetes | [6] |
| 7 | rs10517086 | Type 1 diabetes | [6] |
| 8 | rs1051730 | Nicotine dependence, peripheral arterial disease | [7] |
|  |  | Lung cancer | [8] |
| 9 | rs10778213 | C-reactive protein | [9] |
| 10 | rs10838738 | Body mass index | [10] |
| 11 | rs10958409 | Intracranial aneurysm | [11] |
| 12 | rs1111875 | Type 2 diabetes | [12-14] |
| 13 | rs11203203 | Type 1 diabetes | [6] |
| 14 | rs11206510 | Serum LDL cholesterol | [3, 15] |
|  |  | Myocardial infarction (early onset) | [16] |
| 15 | rs1167998 | Triglycerides | [2] |
| 16 | rs12130333 | Triglycerides | [17] |
| 17 | rs12251307 | Type 1 diabetes | [6] |
| 18 | rs12272004 | Serum LDL cholesterol | [2] |
|  |  | Triglycerides | [2] |
|  |  | Plasma carotenoid and tocopherol levels | [18] |
| 19 | rs12425791 | Stroke | [19] |
| 20 | rs1260326 | Waist circumference and related phenotypes | [20] |
|  |  | Triglycerides | [3] |
|  |  | Other metabolic traits (Triglycerides) | [21] |
| 21 | rs12619285 | Plasma eosinophil count | [22] |
| 22 | rs12670798 | Serum LDL cholesterol | [2] |
| 23 | rs12678919 | Serum HDL cholesterol | [3] |
|  |  | Triglycerides | [3] |
| 24 | rs12970134 | Waist circumference and related phenotypes | [20] |
|  |  | Body mass index | [23] |
|  |  | Weight | [23] |
| 25 | rs13129697 | Uric acid | [24] |
| 26 | rs13194491 | Transferrin saturation | [25] |
| 27 | rs13266634 | Type 2 diabetes | [14] |
| 28 | rs1333040 | Intracranial aneurysm | [11] |
| 29 | rs1378942 | Diastolic Blood Pressure | [26] |
| 30 | rs1387153 | Fasting plasma glucose | [27] |
| 31 | rs1420101 | Plasma eosinophil count | [22] |
| 32 | rs1424233 | Obesity | [28] |
| 33 | rs1465788 | Type 1 diabetes | [6] |
| 34 | rs1532085 | Serum HDL cholesterol | [2, 21] |
| 35 | rs1532624 | Serum HDL cholesterol | [2] |
| 36 | rs157580 | Serum LDL cholesterol | [2] |
|  |  | Alzheimer's disease | [29] |
| 37 | rs1701704 | Type 1 diabetes | [30] |
| 38 | rs17145738 | Triglycerides | [15, 17] |
| 39 | rs17367504 | Systolic blood pressure | [26] |
| 40 | rs174570 | Cholesterol, total | [2] |
|  |  | Serum LDL cholesterol | [2] |
| 41 | rs1746048 | Myocardial infarction (early onset) | [16] |
| 42 | rs17696736 | Type 1 diabetes | [31-33] |
| 43 | rs1780324 | Plasma levels of alkaline phosphatase | [34] |
| 44 | rs1799969 | Soluble ICAM-1 | [35] |
| 45 | rs1800562 | Serum markers of iron status (serum iron, serum transferring, and transferrin saturation) | [25] |
| 46 | rs1800775 | Triglycerides | [12] |
|  |  | Serum HDL cholesterol | [17] |
| 47 | rs1800961 | Serum HDL cholesterol | [3] |
| 48 | rs1864163 | Serum HDL cholesterol | [15] |
| 49 | rs1880887 | Plasma levels of alkaline phosphatase | [36] |
| 50 | rs1892534 | Plasma C-reactive protein | [9] |
| 51 | rs1893217 | Type 1 diabetes | [6] |
| 52 | rs1990760 | Type 1 diabetes | [6, 31] |
| 53 | rs2048327* | Coronary artery disease | [37] |
| 54 | rs2083637 | Serum HDL cholesterol | [2] |
| 55 | rs2200733 | Atrial fibrillation/atrial flutter | [38] |
|  |  | Ischemic stroke | [39] |
| 56 | rs2228671 | Cholesterol, total | [2] |
|  |  | Serum LDL cholesterol | [2] |
| 57 | rs2231142 | Serum urate | [40] |
| 58 | rs2237892 | Type 2 diabetes | [14, 41] |
| 59 | rs2240466 | Triglycerides | [2] |
| 60 | rs2250417 | Serum interleukin-18 levels | [36] |
| 61 | rs2271293 | Serum HDL cholesterol | [2, 3] |
| 62 | rs2290400 | Type 1 diabetes | [6] |
| 63 | rs2383208 | Type 2 diabetes | [14] |
| 64 | rs2384550 | Diastolic blood pressure | [42] |
| 65 | rs2476601 | Type 1 diabetes | [6, 31] |
|  |  | Rheumatoid arthritis | [43] |
| 66 | rs255049 | Serum HDL cholesterol | [21] |
| 67 | rs2568958 | Body mass index | [23] |
|  |  | Weight | [23] |
| 68 | rs2647044 | Type 1 diabetes | [1] |
| 69 | rs2650000 | Serum LDL cholesterol | [3] |
|  |  | Plasma C-reactive protein | [21] |
| 70 | rs2664170 | Type 1 diabetes | [6] |
| 71 | rs2681472 | Diastolic blood pressure | [42] |
|  |  | Hypertension | [42] |
| 72 | rs2681492 | Systolic blood pressure | [42] |
| 73 | rs2722425 | Fasting plasma glucose | [44] |
| 74 | rs2794520 | Plasma C-reactive protein | [45] |
|  |  | Plasma C-reactive protein | [21] |
| 75 | rs281437 | Plasma Soluble ICAM-1 | [35] |
| 76 | rs2903692 | Type 1 diabetes | [1] |
| 77 | rs2967605 | Serum HDL cholesterol | [3] |
| 78 | rs29941 | Body mass index | [23] |
|  |  | Weight | [23] |
| 79 | rs3024505 | Type 1 diabetes | [6] |
| 80 | rs3184504 | Plasma eosinophil count | [22] |
|  |  | Diastolic blood pressure | [42] |
|  |  | Systolic blood pressure | [42] |
|  |  | Type 1 diabetes | [6] |
| 81 | rs3764261 | Serum HDL cholesterol | [15, 21] |
|  |  | Waist circumference and related phenotypes | [20] |
|  |  | Serum LDL cholesterol | [46] |
| 82 | rs3811647 | Serum markers of iron status | [25] |
| 83 | rs3846662 | Cholesterol, total | [2] |
|  |  | Serum LDL cholesterol | [2] |
| 84 | rs3848445 | Plasma free triiodothryonine | [36] |
| 85 | rs3905000 | Serum HDL cholesterol | [2] |
| 86 | rs4129267 | Serum levels of IL-6 soluble receptor | [36] |
| 87 | rs4143832 | Plasma eosinophil count | [22] |
| 88 | rs4149268 | Serum HDL cholesterol | [15] |
| 89 | rs425105 | Type 1 diabetes | [6] |
| 90 | rs439401 | Triglycerides | [2] |
| 91 | rs4402960 | Type 2 diabetes | [12, 13, 47] |
| 92 | rs4505848 | Type 1 diabetes | [6] |
| 93 | rs4607517 | Fasting plasma glucose | [48] |
| 94 | rs4654748 | Folate pathway vitamins | [49] |
| 95 | rs4712523 | Type 2 diabetes | [14] |
| 96 | rs471364 | Serum HDL cholesterol | [3] |
| 97 | rs4763879 | Type 1 diabetes | [6] |
| 98 | rs4788084 | Type 1 diabetes | [6] |
| 99 | rs4796217 | Serum macrophage inflammatory protein-1b | [36] |
| 100 | rs4857855 | Plasma eosinophil count | [22] |
| 101 | rs4900384 | Type 1 diabetes | [6] |
| 102 | rs4939883 | Serum HDL cholesterol | [2] |
|  |  | Cholesterol, total | [2] |
| 103 | rs4977574 | Myocardial infarction (early onset) | [16] |
| 104 | rs5015480 | Type 2 diabetes | [47] |
| 105 | rs505922 | Serum tumor necrosis factor-α | [36] |
|  |  | Venous thromboembolism | [50] |
| 106 | rs5215 | Type 2 diabetes | [47] |
| 107 | rs5498 | Soluble ICAM-1 | [35] |
| 108 | rs560887 | Fasting plasma glucose | [21, 48, 51] |
| 109 | rs5753037 | Type 1 diabetes | [6] |
| 110 | rs602662 | Folate pathway vitamins | [49] |
| 111 | rs6265 | Body mass index | [23] |
| 112 | rs646776 | Serum LDL cholesterol | [2, 17, 21] |
|  |  | Cholesterol, total | [2] |
|  |  | Myocardial infarction (early onset) | [16] |
| 113 | rs6495122 | Diastolic blood pressure | [42] |
| 114 | rs6499640 | Body mass index | [23] |
|  |  | Weight | [23] |
| 115 | rs6511720 | Serum LDL cholesterol | [3, 15, 17] |
| 116 | rs653178 | Diastolic Blood Pressure | [26] |
| 117 | rs6544713 | Serum LDL cholesterol | [3] |
| 118 | rs657152 | Plasma levels of alkaline phosphatase | [34] |
| 119 | rs6711736* | Hypertension (young onset) | [5] |
| 120 | rs6725887 | Myocardial infarction (early onset) | [16] |
| 121 | rs673548 | Triglycerides | [21] |
| 122 | rs6742078 | Serum bilirubin levels | [52] |
| 123 | rs6754295 | Serum HDL cholesterol | [2] |
|  |  | Triglycerides | [2] |
| 124 | rs6756629 | Cholesterol, total | [2] |
|  |  | Serum LDL cholesterol | [2] |
| 125 | rs6919346 | Plasma Lp (a) levels | [53] |
| 126 | rs6922269 | Coronary disease | [54] |
| 127 | rs693 | Serum LDL cholesterol | [2, 17, 21] |
|  |  | Cholesterol, total | [2] |
| 128 | rs6931514 | Type 2 diabetes | [55] |
| 129 | rs700651 | Intracranial aneurysm | [11] |
| 130 | rs7111341 | Type 1 diabetes | [6] |
| 131 | rs7112513 | Protein quantitative trait loci (Soluble transferrin receptor) | [36] |
| 132 | rs7120118 | Serum HDL cholesterol | [21] |
| 133 | rs714052 | Triglycerides | [3] |
| 134 | rs7202877 | Type 1 diabetes | [6] |
| 135 | rs7310409 | Serum C-reactive protein | [9] |
| 136 | rs737267 | Serum urate | [56] |
| 137 | rs7395662 | Serum HDL cholesterol | [2] |
| 138 | rs7498665 | Body mass index | [10, 23] |
|  |  | Weight | [23] |
| 139 | rs7561317 | Body mass index | [23] |
|  |  | Weight | [23] |
| 140 | rs7578597 | Type 2 diabetes | [55] |
| 141 | rs763361 | Type 1 diabetes | [31] |
| 142 | rs7647305 | Body mass index | [23] |
|  |  | Weight | [23] |
| 143 | rs7679 | Serum HDL cholesterol | [3] |
|  |  | Triglycerides | [3] |
| 144 | rs7756992 | Type 2 diabetes | [57] |
| 145 | rs7770628 | Serum lipoprotein A | [36] |
| 146 | rs780094 | Triglycerides | [2], [15, 17] |
|  |  | Serum C-reactive protein | [9] |
| 147 | rs7804356 | Type 1 diabetes | [6] |
| 148 | rs7901695 | Type 2 diabetes | [47] |
| 149 | rs7903146 | Type 2 diabetes | [12-14, 55, 57-59] |
| 150 | rs7961894 | Mean platelet volume | [60] |
| 151 | rs8050136 | Type 2 diabetes | [47, 59] |
|  |  | Body mass index | [23] |
|  |  | Weight | [23] |
| 152 | rs925946 | Body mass index | [23] |
|  |  | Weight | [23] |
| 153 | rs9298506 | Intracranial aneurysm | [11] |
| 154 | rs9388489 | Type 1 diabetes | [6] |
| 155 | rs9467160 | Plasma levels of alkaline phosphatase | [34] |
| 156 | rs947474 | Type 1 diabetes | [33] |
| 157 | rs9976767 | Type 1 diabetes | [61] |
| 158 | rs9989419 | Serum HDL cholesterol | [15] |

**Reference**

1. Hakonarson H, Grant SF, Bradfield JP, Marchand L, Kim CE, Glessner JT, Grabs R, Casalunovo T, Taback SP, Frackelton EC *et al*: **A genome-wide association study identifies KIAA0350 as a type 1 diabetes gene**. *Nature* 2007, **448**(7153):591-594.

2. Aulchenko YS, Ripatti S, Lindqvist I, Boomsma D, Heid IM, Pramstaller PP, Penninx BW, Janssens AC, Wilson JF, Spector T *et al*: **Loci influencing lipid levels and coronary heart disease risk in 16 European population cohorts**. *Nat Genet* 2009, **41**(1):47-55.

3. Kathiresan S, Willer CJ, Peloso GM, Demissie S, Musunuru K, Schadt EE, Kaplan L, Bennett D, Li Y, Tanaka T *et al*: **Common variants at 30 loci contribute to polygenic dyslipidemia**. *Nat Genet* 2009, **41**(1):56-65.

4. Arking DE, Pfeufer A, Post W, Kao WHL, Newton-Cheh C, Ikeda M, West K, Kashuk C, Akyol M, Perz S *et al*: **A common genetic variant in the NOS1 regulator NOS1AP modulates cardiac repolarization**. *Nat Genet* 2006, **38**(6):644--651.

5. Yang HC, Liang YJ, Wu YL, Chung CM, Chiang KM, Ho HY, Ting CT, Lin TH, Sheu SH, Tsai WC *et al*: **Genome-wide association study of young-onset hypertension in the Han Chinese population of Taiwan**. *PLoS One* 2009, **4**(5):e5459.

6. Barrett JC, Clayton DG, Concannon P, Akolkar B, Cooper JD, Erlich HA, Julier C, Morahan G, Nerup J, Nierras C *et al*: **Genome-wide association study and meta-analysis find that over 40 loci affect risk of type 1 diabetes**. *Nat Genet* 2009, **41**:703-707.

7. Thorgeirsson TE, Geller F, Sulem P, Rafnar T, Wiste A, Magnusson KP, Manolescu A, Thorleifsson G, Stefansson H, Ingason A *et al*: **A variant associated with nicotine dependence, lung cancer and peripheral arterial disease**. *Nature* 2008, **452**(7187):638-642.

8. McKay JD, Hung RJ, Gaborieau V, Boffetta P, Chabrier A, Byrnes G, Zaridze D, Mukeria A, Szeszenia-Dabrowska N, Lissowska J *et al*: **Lung cancer susceptibility locus at 5p15.33**. *Nat Genet* 2008, **40**(12):1404-1406.

9. Ridker PM, Pare G, Parker A, Zee RY, Danik JS, Buring JE, Kwiatkowski D, Cook NR, Miletich JP, Chasman DI: **Loci related to metabolic-syndrome pathways including LEPR,HNF1A, IL6R, and GCKR associate with plasma C-reactive protein: the Women's Genome Health Study**. *Am J Hum Genet* 2008, **82**(5):1185-1192.

10. Willer CJ, Speliotes EK, Loos RJ, Li S, Lindgren CM, Heid IM, Berndt SI, Elliott AL, Jackson AU, Lamina C *et al*: **Six new loci associated with body mass index highlight a neuronal influence on body weight regulation**. *Nat Genet* 2009, **41**(1):25-34.

11. Bilguvar K, Yasuno K, Niemela M, Ruigrok YM, von Und Zu Fraunberg M, van Duijn CM, van den Berg LH, Mane S, Mason CE, Choi M *et al*: **Susceptibility loci for intracranial aneurysm in European and Japanese populations**. *Nat Genet* 2008, **40**(12):1472-1477.

12. Saxena R, Voight BF, Lyssenko V, Burtt NP, de Bakker PI, Chen H, Roix JJ, Kathiresan S, Hirschhorn JN, Daly MJ *et al*: **Genome-wide association analysis identifies loci for type 2 diabetes and triglyceride levels**. *Science* 2007, **316**(5829):1331-1336.

13. Scott LJ, Mohlke KL, Bonnycastle LL, Willer CJ, Li Y, Duren WL, Erdos MR, Stringham HM, Chines PS, Jackson AU *et al*: **A genome-wide association study of type 2 diabetes in Finns detects multiple susceptibility variants**. *Science* 2007, **316**(5829):1341-1345.

14. Takeuchi F, Serizawa M, Yamamoto K, Fujisawa T, Nakashima E, Ohnaka K, Ikegami H, Sugiyama T, Katsuya T, Miyagishi M *et al*: **Confirmation of multiple risk Loci and genetic impacts by a genome-wide association study of type 2 diabetes in the Japanese population**. *Diabetes* 2009, **58**(7):1690-1699.

15. Willer CJ, Sanna S, Jackson AU, Scuteri A, Bonnycastle LL, Clarke R, Heath SC, Timpson NJ, Najjar SS, Stringham HM *et al*: **Newly identified loci that influence lipid concentrations and risk of coronary artery disease**. *Nat Genet* 2008, **40**(2):161-169.

16. Kathiresan S, Voight BF, Purcell S, Musunuru K, Ardissino D, Mannucci PM, Anand S, Engert JC, Samani NJ, Schunkert H *et al*: **Genome-wide association of early-onset myocardial infarction with single nucleotide polymorphisms and copy number variants**. *Nat Genet* 2009, **41**(3):334-341.

17. Kathiresan S, Melander O, Guiducci C, Surti A, Burtt NP, Rieder MJ, Cooper GM, Roos C, Voight BF, Havulinna AS *et al*: **Six new loci associated with blood low-density lipoprotein cholesterol, high-density lipoprotein cholesterol or triglycerides in humans**. *Nat Genet* 2008, **40**(2):189-197.

18. Ferrucci L, Perry JR, Matteini A, Perola M, Tanaka T, Silander K, Rice N, Melzer D, Murray A, Cluett C *et al*: **Common variation in the beta-carotene 15,15'-monooxygenase 1 gene affects circulating levels of carotenoids: a genome-wide association study**. *Am J Hum Genet* 2009, **84**(2):123-133.

19. Ikram MA, Seshadri S, Bis JC, Fornage M, DeStefano AL, Aulchenko YS, Debette S, Lumley T, Folsom AR, van den Herik EG *et al*: **Genomewide association studies of stroke**. *N Engl J Med* 2009, **360**(17):1718-1728.

20. Chambers JC, Elliott P, Zabaneh D, Zhang W, Li Y, Froguel P, Balding D, Scott J, Kooner JS: **Common genetic variation near MC4R is associated with waist circumference and insulin resistance**. *Nat Genet* 2008, **40**(6):716-718.

21. Sabatti C, Service SK, Hartikainen AL, Pouta A, Ripatti S, Brodsky J, Jones CG, Zaitlen NA, Varilo T, Kaakinen M *et al*: **Genome-wide association analysis of metabolic traits in a birth cohort from a founder population**. *Nat Genet* 2009, **41**(1):35-46.

22. Gudbjartsson DF, Bjornsdottir US, Halapi E, Helgadottir A, Sulem P, Jonsdottir GM, Thorleifsson G, Helgadottir H, Steinthorsdottir V, Stefansson H *et al*: **Sequence variants affecting eosinophil numbers associate with asthma and myocardial infarction**. *Nat Genet* 2009, **41**(3):342-347.

23. Thorleifsson G, Walters GB, Gudbjartsson DF, Steinthorsdottir V, Sulem P, Helgadottir A, Styrkarsdottir U, Gretarsdottir S, Thorlacius S, Jonsdottir I *et al*: **Genome-wide association yields new sequence variants at seven loci that associate with measures of obesity**. *Nat Genet* 2009, **41**(1):18-24.

24. Zemunik T, Boban M, Lauc G, Jankovic S, Rotim K, Vatavuk Z, Bencic G, Dogas Z, Boraska V, Torlak V *et al*: **Genome-wide association study of biochemical traits in Korcula Island, Croatia**. *Croat Med J* 2009, **50**(1):23-33.

25. Benyamin B, McRae AF, Zhu G, Gordon S, Henders AK, Palotie A, Peltonen L, Martin NG, Montgomery GW, Whitfield JB *et al*: **Variants in TF and HFE explain approximately 40% of genetic variation in serum-transferrin levels**. *Am J Hum Genet* 2009, **84**(1):60-65.

26. Newton-Cheh C, Johnson T, Gateva V, Tobin MD, Bochud M, Coin L, Najjar SS, Zhao JH, Heath SC, Eyheramendy S *et al*: **Genome-wide association study identifies eight loci associated with blood pressure**. *Nat Genet* 2009, **41**:666-676.

27. Bouatia-Naji N, Bonnefond A, Cavalcanti-Proenca C, Sparso T, Holmkvist J, Marchand M, Delplanque J, Lobbens S, Rocheleau G, Durand E *et al*: **A variant near MTNR1B is associated with increased fasting plasma glucose levels and type 2 diabetes risk**. *Nat Genet* 2009, **41**(1):89-94.

28. Meyre D, Delplanque J, Chevre JC, Lecoeur C, Lobbens S, Gallina S, Durand E, Vatin V, Degraeve F, Proenca C *et al*: **Genome-wide association study for early-onset and morbid adult obesity identifies three new risk loci in European populations**. *Nat Genet* 2009, **41**(2):157-159.

29. Feulner TM, Laws SM, Friedrich P, Wagenpfeil S, Wurst SH, Riehle C, Kuhn KA, Krawczak M, Schreiber S, Nikolaus S *et al*: **Examination of the current top candidate genes for AD in a genome-wide association study**. *Mol Psychiatry* 2009.

30. Hakonarson H, Qu HQ, Bradfield JP, Marchand L, Kim CE, Glessner JT, Grabs R, Casalunovo T, Taback SP, Frackelton EC *et al*: **A novel susceptibility locus for type 1 diabetes on Chr12q13 identified by a genome-wide association study**. *Diabetes* 2008, **57**(4):1143-1146.

31. Todd JA, Walker NM, Cooper JD, Smyth DJ, Downes K, Plagnol V, Bailey R, Nejentsev S, Field SF, Payne F *et al*: **Robust associations of four new chromosome regions from genome-wide analyses of type 1 diabetes**. *Nat Genet* 2007, **39**(7):857-864.

32. WTCCC: **Genome-wide association study of 14,000 cases of seven common diseases and 3,000 shared controls**. *Nature* 2007, **447**(7145):661-678.

33. Cooper JD, Smyth DJ, Smiles AM, Plagnol V, Walker NM, Allen JE, Downes K, Barrett JC, Healy BC, Mychaleckyj JC *et al*: **Meta-analysis of genome-wide association study data identifies additional type 1 diabetes risk loci**. *Nat Genet* 2008, **40**(12):1399-1401.

34. Yuan X, Waterworth D, Perry JR, Lim N, Song K, Chambers JC, Zhang W, Vollenweider P, Stirnadel H, Johnson T *et al*: **Population-based genome-wide association studies reveal six loci influencing plasma levels of liver enzymes**. *Am J Hum Genet* 2008, **83**(4):520-528.

35. Pare G, Chasman DI, Kellogg M, Zee RY, Rifai N, Badola S, Miletich JP, Ridker PM: **Novel association of ABO histo-blood group antigen with soluble ICAM-1: results of a genome-wide association study of 6,578 women**. *PLoS Genet* 2008, **4**(7):e1000118.

36. Melzer D, Perry JR, Hernandez D, Corsi AM, Stevens K, Rafferty I, Lauretani F, Murray A, Gibbs JR, Paolisso G *et al*: **A genome-wide association study identifies protein quantitative trait loci (pQTLs)**. *PLoS Genet* 2008, **4**(5):e1000072.

37. Tregouet DA, Konig IR, Erdmann J, Munteanu A, Braund PS, Hall AS, Grosshennig A, Linsel-Nitschke P, Perret C, DeSuremain M *et al*: **Genome-wide haplotype association study identifies the SLC22A3-LPAL2-LPA gene cluster as a risk locus for coronary artery disease**. *Nat Genet* 2009, **41**(3):283-285.

38. Gudbjartsson DF, Arnar DO, Helgadottir A, Gretarsdottir S, Holm H, Sigurdsson A, Jonasdottir A, Baker A, Thorleifsson G, Kristjansson K *et al*: **Variants conferring risk of atrial fibrillation on chromosome 4q25**. *Nature* 2007, **448**(7151):353-357.

39. Gretarsdottir S, Thorleifsson G, Manolescu A, Styrkarsdottir U, Helgadottir A, Gschwendtner A, Kostulas K, Kuhlenbaumer G, Bevan S, Jonsdottir T *et al*: **Risk variants for atrial fibrillation on chromosome 4q25 associate with ischemic stroke**. *Ann Neurol* 2008, **64**(4):402-409.

40. Dehghan A, Kottgen A, Yang Q, Hwang SJ, Kao WL, Rivadeneira F, Boerwinkle E, Levy D, Hofman A, Astor BC *et al*: **Association of three genetic loci with uric acid concentration and risk of gout: a genome-wide association study**. *Lancet* 2008, **372**(9654):1953-1961.

41. Yasuda K, Miyake K, Horikawa Y, Hara K, Osawa H, Furuta H, Hirota Y, Mori H, Jonsson A, Sato Y *et al*: **Variants in KCNQ1 are associated with susceptibility to type 2 diabetes mellitus**. *Nat Genet* 2008(40):1092-1097.

42. Levy D, Ehret GB, Rice K, Verwoert GC, Launer LJ, Dehghan A, Glazer NL, Morrison AC, Johnson AD, Aspelund T *et al*: **Genome-wide association study of blood pressure and hypertension**. *Nat Genet* 2009.

43. Plenge RM, Seielstad M, Padyukov L, Lee AT, Remmers EF, Ding B, Liew A, Khalili H, Chandrasekaran A, Davies LR *et al*: **TRAF1-C5 as a risk locus for rheumatoid arthritis--a genomewide study**. *N Engl J Med* 2007, **357**(12):1199-1209.

44. Meigs JB, Manning AK, Fox CS, Florez JC, Liu C, Cupples LA, Dupuis J: **Genome-wide association with diabetes-related traits in the Framingham Heart Study**. *BMC Med Genet* 2007, **8 Suppl 1**:S16.

45. Benjamin EJ, Dupuis J, Larson MG, Lunetta KL, Booth SL, Govindaraju DR, Kathiresan S, Keaney JF, Jr., Keyes MJ, Lin JP *et al*: **Genome-wide association with select biomarker traits in the Framingham Heart Study**. *BMC Med Genet* 2007, **8 Suppl 1**:S11.

46. Hiura Y, Shen CS, Kokubo Y, Okamura T, Morisaki T, Tomoike H, Yoshida T, Sakamoto H, Goto Y, Nonogi H *et al*: **Identification of genetic markers associated with high-density lipoprotein-cholesterol by genome-wide screening in a Japanese population**. *Circ J* 2009, **73**(6):1119-1126.

47. Zeggini E, Weedon MN, Lindgren CM, Frayling TM, Elliott KS, Lango H, Timpson NJ, Perry JR, Rayner NW, Freathy RM *et al*: **Replication of genome-wide association signals in UK samples reveals risk loci for type 2 diabetes**. *Science* 2007, **316**(5829):1336-1341.

48. Prokopenko I, Langenberg C, Florez JC, Saxena R, Soranzo N, Thorleifsson G, Loos RJ, Manning AK, Jackson AU, Aulchenko Y *et al*: **Variants in MTNR1B influence fasting glucose levels**. *Nat Genet* 2009, **41**(1):77-81.

49. Tanaka T, Scheet P, Giusti B, Bandinelli S, Piras MG, Usala G, Lai S, Mulas A, Corsi AM, Vestrini A *et al*: **Genome-wide association study of vitamin B6, vitamin B12, folate, and homocysteine blood concentrations**. *Am J Hum Genet* 2009, **84**(4):477-482.

50. Tregouet DA, Heath S, Saut N, Biron-Andreani C, Schved JF, Pernod G, Galan P, Drouet L, Zelenika D, Juhan-Vague I *et al*: **Common susceptibility alleles are unlikely to contribute as strongly as the FV and ABO loci to VTE risk: results from a GWAS approach**. *Blood* 2009, **113**(21):5298-5303.

51. Bouatia-Naji N, Rocheleau G, Van Lommel L, Lemaire K, Schuit F, Cavalcanti-Proenca C, Marchand M, Hartikainen AL, Sovio U, De Graeve F *et al*: **A polymorphism within the G6PC2 gene is associated with fasting plasma glucose levels**. *Science* 2008, **320**(5879):1085-1088.

52. Johnson AD, Kavousi M, Smith AV, Chen MH, Dehghan A, Aspelund T, Lin JP, van Duijn CM, Harris TB, Cupples LA *et al*: **Genome-wide association meta-analysis for total serum bilirubin levels**. *Hum Mol Genet* 2009, **18**(14):2700-2710.

53. Ober C, Nord AS, Thompson EE, Pan L, Tan Z, Cusanovich D, Sun Y, Nicolae R, Edelstein C, Schneider DH *et al*: **Genome-wide association study of plasma lipoprotein(a) levels identifies multiple genes on chromosome 6q**. *J Lipid Res* 2009, **50**(5):798-806.

54. Samani NJ, Erdmann J, Hall AS, Hengstenberg C, Mangino M, Mayer B, Dixon RJ, Meitinger T, Braund P, Wichmann HE *et al*: **Genomewide association analysis of coronary artery disease**. *N Engl J Med* 2007, **357**(5):443-453.

55. Zeggini E, Scott LJ, Saxena R, Voight BF, Marchini JL, Hu T, de Bakker PI, Abecasis GR, Almgren P, Andersen G *et al*: **Meta-analysis of genome-wide association data and large-scale replication identifies additional susceptibility loci for type 2 diabetes**. *Nat Genet* 2008, **40**(5):638-645.

56. Vitart V, Rudan I, Hayward C, Gray NK, Floyd J, Palmer CN, Knott SA, Kolcic I, Polasek O, Graessler J *et al*: **SLC2A9 is a newly identified urate transporter influencing serum urate concentration, urate excretion and gout**. *Nat Genet* 2008, **40**(4):437-442.

57. Steinthorsdottir V, Thorleifsson G, Reynisdottir I, Benediktsson R, Jonsdottir T, Walters GB, Styrkarsdottir U, Gretarsdottir S, Emilsson V, Ghosh S *et al*: **A variant in CDKAL1 influences insulin response and risk of type 2 diabetes**. *Nat Genet* 2007, **39**(6):770-775.

58. Sladek R, Rocheleau G, Rung J, Dina C, Shen L, Serre D, Boutin P, Vincent D, Belisle A, Hadjadj S *et al*: **A genome-wide association study identifies novel risk loci for type 2 diabetes**. *Nature* 2007, **445**(7130):881-885.

59. Timpson NJ, Lindgren CM, Weedon MN, Randall J, Ouwehand WH, Strachan DP, Rayner NW, Walker M, Hitman GA, Doney AS *et al*: **Adiposity-related heterogeneity in patterns of type 2 diabetes susceptibility observed in genome-wide association data**. *Diabetes* 2009, **58**(2):505-510.

60. Meisinger C, Prokisch H, Gieger C, Soranzo N, Mehta D, Rosskopf D, Lichtner P, Klopp N, Stephens J, Watkins NA *et al*: **A genome-wide association study identifies three loci associated with mean platelet volume**. *Am J Hum Genet* 2009, **84**(1):66-71.

61. Grant SF, Qu HQ, Bradfield JP, Marchand L, Kim CE, Glessner JT, Grabs R, Taback SP, Frackelton EC, Eckert AW *et al*: **Follow-up analysis of genome-wide association data identifies novel loci for type 1 diabetes**. *Diabetes* 2009, **58**(1):290-295.
