# Supplementary material for: Geographic differences in allele frequencies of susceptibility SNPs for cardiovascular disease
Source: BMC Med Genet. 2011 Apr 20;12:55. doi: 10.1186/1471-2350-12-55 (PMC3103418; doi:10.1186/1471-2350-12-55)
Supplement: Additional file 4 — Table S3. The number of risk alleles with various categories of RAF differences: comparisons of a given geographic area versus the rest of the world. [file 1471-2350-12-55-S4.DOC]

**Table S3**. The number of risk alleles with various categories of RAF differences: comparisons of a given geographic area versus the rest of the world.

| Comparison between Geographic areas | *ΔF*<-0.3 | *ΔF*<-0.2 | *ΔF*<-0.1 | -0.1≤*ΔF*≤0.1 | *ΔF*>0.1 | *ΔF*>0.2 | *ΔF*>0.3 |
| --- | --- | --- | --- | --- | --- | --- | --- |
| AFRICA vs. Non-AFRICA | 12 (12) | 28 (28) | 54 (53) | 59 (17) | 45 (43) | 23 (23) | 10 (10) |
| MIDDLE_EAST vs. Non-MIDDLE_EAST | 1 (1) | 11 (10) | 36 (22) | 96 (6) | 26 (14) | 2 (2) | 1 (1) |
| EUROPE vs. Non-EUROPE | 4 (4) | 11 (11) | 33 (28) | 98 (13) | 27 (26) | 7 (7) | 1 (1) |
| CENTRAL_SOUTH_ASIA vs. Non-CENTRAL_SOUTH_ASIA | 0 (0) | 0 (0) | 14 (12) | 131 (8) | 13 (11) | 0 (0) | 0 (0) |
| EAST_ASIA vs. Non-EAST_ASIA | 7 (7) | 14 (14) | 41 (41) | 68 (31) | 49 (49) | 33 (33) | 8 (8) |
| AMERICA vs. Non-AMERICA | 12 (12) | 24 (24) | 52 (49) | 57 (13) | 48 (39) | 23 (23) | 9 (9) |
| OCEANIA | 19 (19) | 30 (26) | 47 (30) | 54 (3) | 57 (30) | 26 (18) | 10 (10) |

The number in parenthesis shows the number of risk alleles with a *ΔF* that is significantly larger (or less) than expected by chance.
