# Supplementary material for: Geographic differences in allele frequencies of susceptibility SNPs for cardiovascular disease
Source: BMC Med Genet. 2011 Apr 20;12:55. doi: 10.1186/1471-2350-12-55 (PMC3103418; doi:10.1186/1471-2350-12-55)
Supplement: Additional file 5 — Table S4. A list of SNPs with significantly higher global FST (Pcor < 0.05) [file 1471-2350-12-55-S5.DOC]

**Table S4**. A list of SNPs with significantly higher global *F*_ST_ (*Pcor* < 0.05)

| SNPs | Trait | Gene | RAF | Global *F*_ST_ | *P* value | *P_cor_* |
| --- | --- | --- | --- | --- | --- | --- |
| rs17696736 | Type 1 diabetes[35-37] | *NAA25* | 0.167 (G) | 0.200 | 0.052 | 0.033 |
| rs2237892 | Type 2 diabetes[45] [18] | *KCNQ1* | 0.820 (C) | 0.198 | 0.055 | 0.035 |
| rs7578597 | Type 2 diabetes[59] | *THADA* | 0.899 (T) | 0.200 | 0.052 | 0.033 |
| rs673548 | Triglycerides[25] | *APOB* | 0.419 (A) | 0.194 | 0.061 | 0.047 |

RAF, risk allele frequency
