# Supplementary material for: Geographic differences in allele frequencies of susceptibility SNPs for cardiovascular disease
Source: BMC Med Genet. 2011 Apr 20;12:55. doi: 10.1186/1471-2350-12-55 (PMC3103418; doi:10.1186/1471-2350-12-55)

**Figure S1.** − Pairwise comparison of population differentiation for SNPs with a significantly higher global *F*_ST_ among 52 populations. The shaded boxes in the matrices indicate the significance level of *F*_ST_ based on the empirical distribution of the 2,036 SNPs for each pair of populations. The inserted subplot shows the comparison for the seven populations based on the geographic areas.


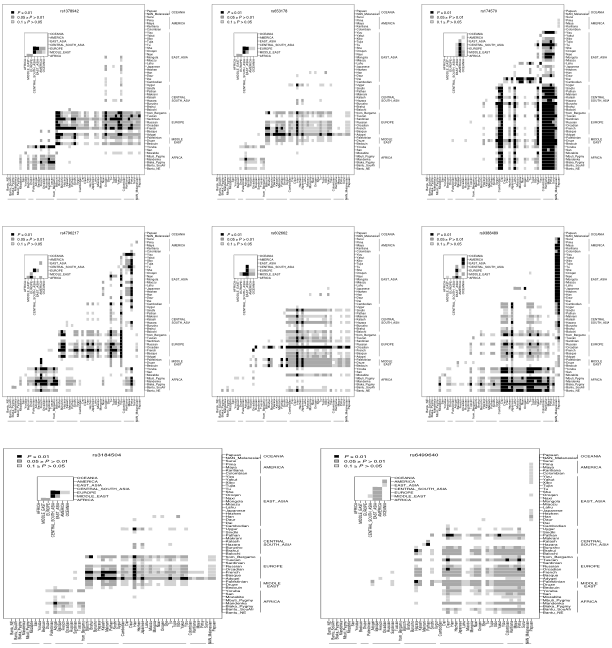

Supplement: Additional file 6 — Figure S1. Pairwise comparison of population differentiation for SNPs with a significantly higher global FST among 52 populations. The shaded boxes in the matrices indicate the significance level of FST based on the empirical distribution of the 2,036 SNPs for each pair of populations. The inserted subplot shows the comparison for the seven populations based on the geographic areas. [file 1471-2350-12-55-S6.DOC]
