# Supplementary material for: Geographic differences in allele frequencies of susceptibility SNPs for cardiovascular disease
Source: BMC Med Genet. 2011 Apr 20;12:55. doi: 10.1186/1471-2350-12-55 (PMC3103418; doi:10.1186/1471-2350-12-55)

**Figure S2**. − Number of SNPs that showed a significantly higher *F*_ST_ in pairwise comparisons among the seven geographic areas.


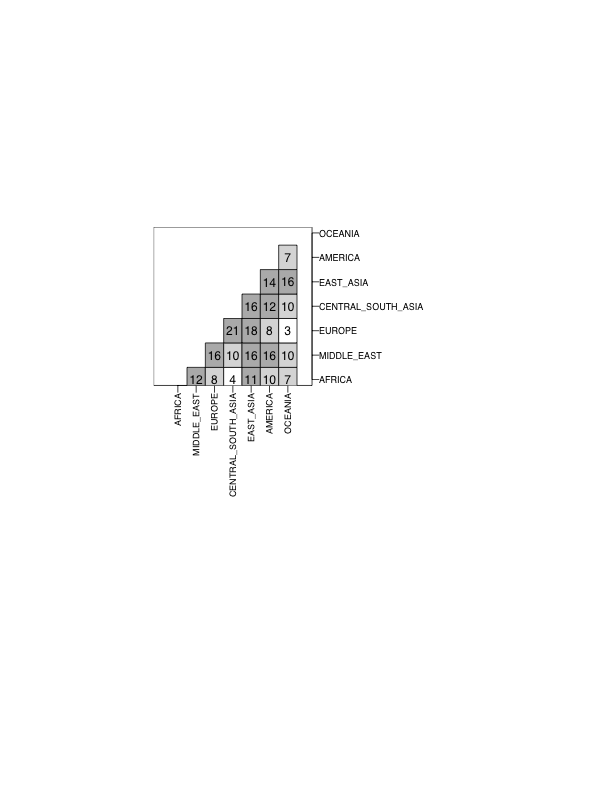

Supplement: Additional file 7 — Figure S2. Number of SNPs that showed a significantly higher FST in pairwise comparisons among the seven geographic areas. [file 1471-2350-12-55-S7.DOC]
