# Supplementary material for: Geographic differences in allele frequencies of susceptibility SNPs for cardiovascular disease
Source: BMC Med Genet. 2011 Apr 20;12:55. doi: 10.1186/1471-2350-12-55 (PMC3103418; doi:10.1186/1471-2350-12-55)
Supplement: Additional file 8 — Figure S3. Multidimensional scaling analysis plot (dimension I/II) of 938 individuals from seven geographic areas using 158 SNPs sampled from the HGDP database. Description: The population 'Mozabite' (black circle) was clustered into the 'Middle East', and 'Europe', which was geographically located in North Africa. The populations in 'Central_South_Asia' showed a cline from 'Middle East' and 'Europe' to 'East_Asia'. Different colors and point characters indicate seven geographic areas (see figure legend). [file 1471-2350-12-55-S8.DOC]

**Figure S3.** Multidimensional scaling analysis plot (dimension I/II) of 938 individuals from seven geographic areas using 158 SNPs sampled from the HGDP database. The population ‘Mozabite’ (black circle) was clustered into the ‘MIDDLE_EAST’, and ‘EUROPE’, which was geographically located in North Africa. The populations in ‘CENTRAL_SOUTH_ASIA’ showed a cline from ‘MIDDLE_EAST’ and ‘EUROPE’ to ‘EAST_ASIA’. Different colors and point characters indicate seven geographic areas (see figure legend).

**
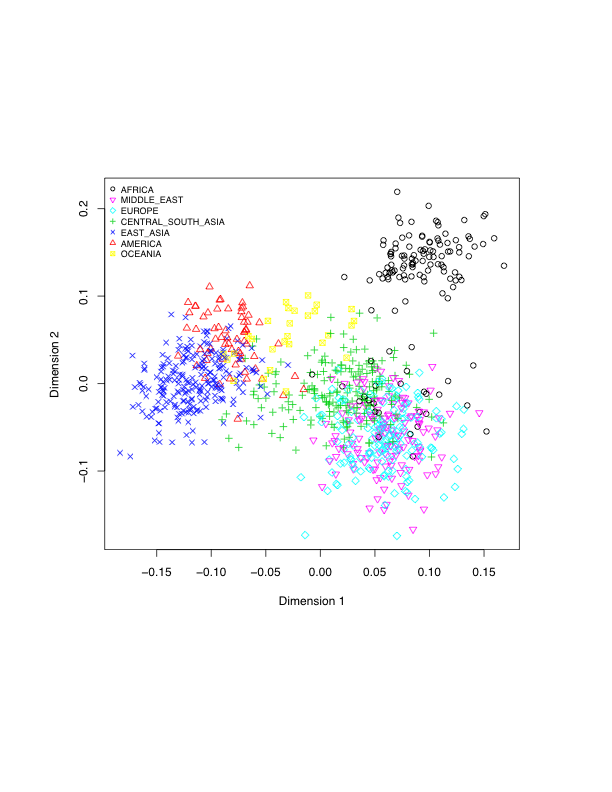
**
