# Supplementary material for: Geographic differences in allele frequencies of susceptibility SNPs for cardiovascular disease
Source: BMC Med Genet. 2011 Apr 20;12:55. doi: 10.1186/1471-2350-12-55 (PMC3103418; doi:10.1186/1471-2350-12-55)
Supplement: Additional file 9 — Table S5. Analysis of molecular variance (AMOVA) of 158 SNPs. [file 1471-2350-12-55-S9.DOC]

**Table S5.** Analysis of molecular variance (AMOVA) of 158 SNPs

| Source | SSD | MSD | d.f. | Variance | % Variation |
| --- | --- | --- | --- | --- | --- |
| Among geographic regions | 11.44 | 1.91 | 6 | 0.014 | 28.6% |
| Among populations within geographic regions | 3.50 | 0.08 | 45 | 0.009 | 18.4% |
| Among individuals within populations | 23.09 | 0.03 | 886 | 0.026 | 53.0% |
| Total | 38.03 | 0.04 | 937 | 0.049 | 100% |

SSD: Sum of squared difference; MSD: Mean square deviations; d.f., degree of freedom
